# Southern hemisphere eastern boundary upwelling systems emerging as future marine heatwave hotspots under greenhouse warming

**DOI:** 10.1038/s41467-022-35666-8

**Published:** 2023-01-03

**Authors:** Shengpeng Wang, Zhao Jing, Lixin Wu, Shantong Sun, Qihua Peng, Hong Wang, Yu Zhang, Jian Shi

**Affiliations:** 1 Frontiers Science Center for Deep Ocean Multispheres and Earth System and Key Laboratory of Physical Oceanography, Ocean University of China, Qingdao, China; 2 Laoshan Laboratory, Qingdao, China; 3 Department of Earth, Ocean and Atmospheric Science, Florida State University, Tallahassee, FL USA; 4 Scripps Institution of Oceanography, University of California San Diego, La Jolla, CA USA; 5 College of Oceanic and Atmospheric Sciences, Ocean University of China, Qingdao, China

**Keywords:** Physical oceanography, Projection and prediction

## Abstract

Marine heatwaves (MHWs) exert devastating impacts on ecosystems and have been revealed to increase in their incidence, duration, and intensity in response to greenhouse warming. The biologically productive eastern boundary upwelling systems (EBUSs) are generally regarded as thermal refugia for marine species due to buffering effects of upwelling on ocean warming. However, using an ensemble of state-of-the-art high-resolution global climate simulations under a high carbon emission scenario, here we show that the MHW stress, measured as the annual cumulative intensity of MHWs, is projected to increase faster in the Southern Hemisphere EBUSs (Humboldt and Benguela current systems) than in their adjacent oceans. This is mainly because the additional warming caused by the weakened eastern boundary currents overwhelms the buffering effect of upwelling. Our findings suggest that the Southern Hemisphere EBUSs will emerge as local hotspots of MHWs in the future, potentially causing severe threats to the ecosystems.

## Abstract (web summary)

The biologically productive eastern boundary upwelling systems (EBUSs) are regarded as thermal refugia in a warming climate. However, the authors here show that Southern Hemisphere EBUSs are likely to become hotspots of future marine heatwaves.

## Introduction

Most parts of the surface ocean have undergone substantial warming during the past several decades as a result of anthropogenic greenhouse gas emissions^1,2^. Along with this mean-state warming, prolonged extreme warm water events, known as marine heatwaves (MHWs)^3^, have increased significantly in their incidence, duration, and intensity on a global scale, as revealed by satellite observations^4^. MHWs often have more adverse ecological and socioeconomic consequences than the gradual increase in the mean-state sea surface temperature (SST) due to the limited capacity of marine species in adjusting to the MHW-triggered abrupt and substantial environmental changes^5^. Although many efforts have been made to evaluate the response of the MHWs in the open ocean to greenhouse warming^5–7^, there is still limited knowledge of long-term MHW changes in the coastal regions where the ecosystems are richest^8–11^ and global drivers of SST changes are modified by local atmospheric and oceanic circulation patterns^12–17^. Global-scale analyses tend to overlook or mask the MHW changes in the coastal regions due to their small occupied area^5–7^.

**Fig. 1:**
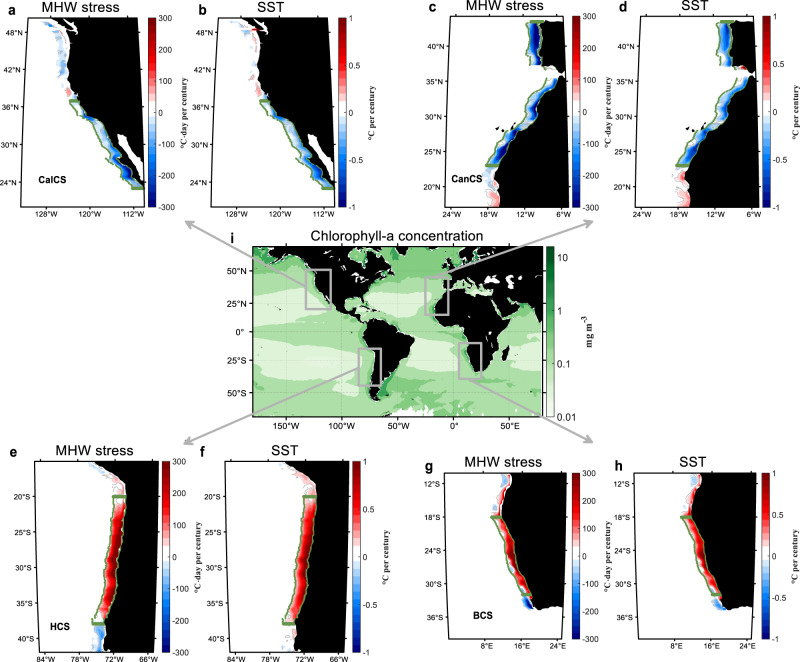
Changes of marine heatwave (MHW) stress and mean-state sea surface temperature (SST) in the eastern boundary upwelling systems (EBUSs) under greenhouse warming projected by CESM-H. The linear trend of MHW stress during 2001–2100 in California current system (CalCS, **a**), Canary current system (CanCS, **c**), Humboldt current system (HCS, **e**) and Benguela current system (BCS, **g**) minus its counterpart in the adjacent ocean (Supplementary Fig. 10), i.e., the coastal and oceanic trend difference (COTD) of MHW stress. **b, d, f, h**, Same as **a, c, e, g**, but for the COTD of annual mean SST. Regions with the COTD insignificant at a 95% confidence level are masked by white. **i**, Geographical location of the four major EBUSs with the shading denoting the satellite-measured mean sea surface chlorophyll-a concentration during 2002–2022.

Located along the eastern boundaries of the Pacific and Atlantic basins, the eastern boundary upwelling systems (EBUSs), including California (CalCS), Canary (CanCS), Humboldt (HCS), and Benguela current systems (BCS), are among the most biologically productive regions around the world^8–11^ (Fig. 1). Equatorward alongshore winds over the EBUSs drive intense upwelling and pump cold and nutrient-rich subsurface water into the euphotic layer by pushing surface water offshore^10^, which plays a pivotal role in sustaining the primary production and biological diversity^18–20^. In addition, the upwelled cold subsurface water is suggested to buffer the SST increase under greenhouse warming^12–17^. This argument is supported by the slower mean-state warming and MHW statistics increase in the EBUSs than in the adjacent ocean, according to the satellite observations during the past several decades, leading the community to hypothesize that the EBUSs will serve as thermal refugia in a warming climate^12–17,21,22^.

However, the limited length of observational records and inherent bias of satellite SST products in the coastal upwelling regions^23,24^ raise concerns on whether the observed slower increases of mean-state SST and MHW statistics in the EBUSs than in the adjacent ocean are the forced response to greenhouse warming or natural multi-decadal variability. In fact, there is some evidence^25–28^ that the anthropogenic change in upwelling, a key process underpinning the thermal refugia of EBUSs, may not emerge until the second half of the twenty-first century. Alternatively, the long-term climate model simulations can provide an opportunity to evaluate the response of MHWs in the EBUSs to greenhouse warming. However, the most recent generation of coupled global climate models (CGCMs) is generally too coarse to resolve the essential atmospheric and oceanic dynamics in the EBUSs^29–33^. In particular, the poorly resolved coastal upwelling by low-resolution CGCMs in the Coupled Model Intercomparison Project Phase 6 (CMIP6)^34^ causes a large warm bias in the mean-state SST that may be further biased into MHWs (Supplementary Table 1; Supplementary Figs. 1 and 2), degrading the fidelity of these CGCMs in projecting MHW changes in the EBUSs under greenhouse warming.

In this study, we evaluate the future change of MHWs in the four major EBUSs under a high carbon emission scenario using an unprecedented long-term high-resolution climate simulation^35^ based on the Community Earth System Model (denoted as CESM-H for short; see “CESM-H” in “Methods”). CESM-H has an ocean resolution of 0.1°, fine enough to resolve the prominent coastal upwelling in the EBUSs. It shows a much-improved representation of present-day mean-state SST and MHW stress (See “Computation of MHW stress” in “Methods”) compared to the low-resolution CMIP6 CGCMs (Supplementary Figs. 1 and 2), giving us confidence in its reliability in projecting MHW changes in the EBUSs. According to the CESM-H’s projection, the Southern Hemisphere EBUSs will likely to become local hotspots of MHWs rather than thermal refugia in the future, as the buffering effect of upwelling is overwhelmed by the additional warming caused by the weakened eastern boundary currents.

## Results

### Projected MHW changes in the EBUSs under a high carbon emission scenario by high-resolution CGCMs

Figure 1a, c, e, g displays the projected linear trends of MHW stress in the EBUSs during 2001–2100 minus their oceanic counterparts, hereinafter referred to as the coastal and oceanic trend difference (COTD) of MHW stress (see “Definition of coastal and oceanic difference” in Methods). For the Northern Hemisphere EBUSs (CalCS and CanCS), there is a significant slowdown of the MHW stress increase, consistent with the thermal refugia hypothesis. On the contrary, the MHW stress exhibits a faster increase over a large fraction of the Southern Hemisphere EBUSs (HCS and BCS), suggesting that the Southern Hemisphere EBUSs would become local hotspots of MHWs in the future under greenhouse warming.

The spatial distributions of COTD of MHW stress in the four EBUSs are strongly correlated with those of mean-state SST (Fig. 1a–h). Their spatial correlation coefficients are above 0.9 for all the EBUSs, suggesting that the suppressed (enlarged) MHW stress increase in some EBUS may be due to the slower (faster) mean-state warming. To further demonstrate this point, we recompute the MHWs defined relative to a contemporaneous equilibrium state^36^ (see “Computation of MHW stress” in “Methods”). Consistent with our argument, the COTD of MHW stress in the four EBUSs reduces to a negligible level once the effect of long-term mean-state SST change on MHW change is removed (Supplementary Fig. 3).

To test whether the COTD of mean-state SST in the EBUSs projected by CESM-H is robust, we compare the CESM-H’s results with those from the high-resolution CGCMs in CMIP6, which have an ocean resolution of at least 0.25° and can partially resolve the coastal upwelling (Supplementary Tables 1 and 2). Because most of the high-resolution CMIP6 CGCM simulations end in 2050 due to their large computational burden^37^, here we compare the COTD among the high-resolution CGCMs, including CESM-H, over 2001–2050. As revealed by CESM-H, the difference in the mean-state warming rates between the EBUSs and their adjacent oceans will have already emerged by 2050 (Supplementary Fig. 4).

**Fig. 2:**
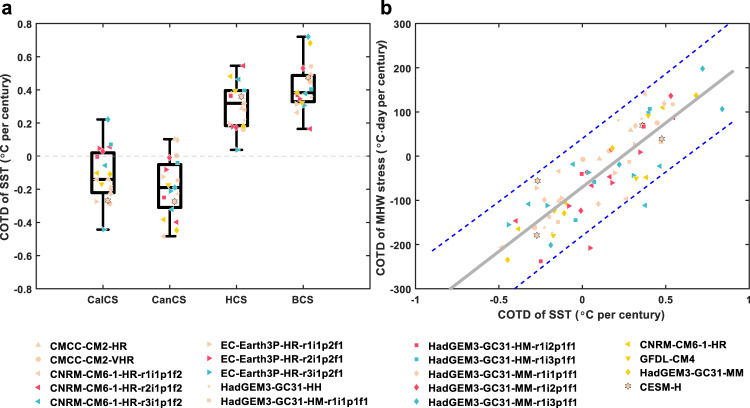
Comparison of the projected marine heatwave (MHW) stress and mean-state sea surface temperature (SST) changes in the eastern boundary upwelling systems (EBUSs) under greenhouse warming between CESM-H and high-resolution coupled global climate models (CGCMs) in CMIP6. **a** Boxplots of coastal and oceanic trend difference (COTD) of mean-state SST in the individual CGCMs. **b** Scatterplot of COTD of MHW stress vs. COTD of mean-state SST in the individual CGCMs. The solid gray line represents the linear regression line, with the blue dashed lines corresponding to its 95% confidence interval. All the COTD values are the spatial average over the regions delineated by the green boxes in Fig. 1a–h.

Figure 2a displays the COTD of mean-state SST projected by individual high-resolution CGCMs, averaged over a subdomain of the EBUSs where the COTD in CESM-H is statistically significant and spatially coherent (delineated by green boxes in Fig. 1a–h). Most of the high-resolution CGCMs in CMIP6 show qualitatively consistent projections with CESM-H. Furthermore, the high-resolution CGCMs projecting a faster (slower) coastal mean-state warming generally exhibit an enlarged (suppressed) coastal MHW stress increase (Fig. 2b). The inter-model correlation coefficient between the regional mean COTDs of mean-state SST and MHW stress reaches up to 0.86, statistically significant at 99% confidence level. This provides further evidence that the slower mean-state warming in the Northern Hemisphere EBUSs would drive suppressed MHW stress increase under greenhouse warming, whereas the opposite is true for the Southern Hemisphere EBUSs. Nevertheless, there is a noticeable inter-model spread of the relation between the COTDs of mean-state SST and MHW stress (Fig. 2b), suggesting that the influence of mean-state warming on MHW stress increase is quantitatively model-dependent and likely to rely on the model’s skill in representing higher-order SST statistics (e.g., variance and skewness) at present as well as their future changes.

### Underlying dynamics for the faster mean-state SST warming in the Southern Hemisphere EBUSs

To understand the underlying dynamics responsible for the different mean-state SST warming rates between the EBUSs and their adjacent oceans, we perform a heat budget analysis for the upper 50-m water column over the EBUSs based on CESM-H (see “Heat budget analysis” in ”Methods”). Here the temperature averaged in the upper 50 m is used as a proxy for SST because its COTD is consistent with that of SST (Supplementary Fig. 5). To distinguish the role of coastal dynamics from large-scale processes in driving mean-state SST change in the EBUSs, each term in the heat budget discussed hereinafter is calculated as the difference from its value in the adjacent ocean.

**Fig. 3:**
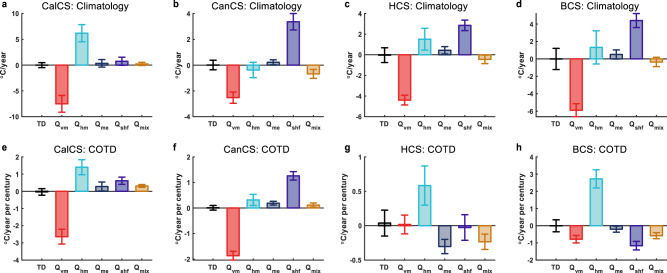
Dynamical processes responsible for the different mean-state sea surface temperature (SST) changes between the eastern boundary upwelling systems (EBUSs) and adjacent oceans under greenhouse warming. Climatological mean (1950–2000) heat budget in the upper 50 m averaged over California current system (CalCS, **a**), Canary current system (CanCS, **b**), Humboldt current system (HCS, **c**) and Benguela current system (BCS, **d**) minus its oceanic counterpart, where TD is the temperature tendency, *Q*_vm_ (*Q*_hm_) the vertical (horizontal) advection by mean flows, *Q*_me_ the temperature flux convergence by mesoscale eddies, *Q*_shf_ the contribution by the net sea surface heat flux and *Q*_mix_ the parameterized subgrid-scale mixing. Regions used for spatial average are delineated by the green boxes in Fig. 1a–h. The error bar denotes the 95% confidence level. **e**–**h**, Same as **a**–**d**, but for the coastal and oceanic trend difference (COTD) of individual heat budget terms during 2001–2100.

For the climatological mean (1950–2000) heat budget (Fig. 3a–d), the vertical advection by mean flows (i.e., upwelling) ${Q}_{{{{{{\rm{vm}}}}}}}$ plays a dominant role in cooling the surface EBUSs. This cooling effect is largely balanced by a net downward sea surface heat flux in CanCS, HCS, and BCS; but is balanced by the mean-flow horizontal advection ${Q}_{{{{{{\rm{hm}}}}}}}$ in CalCS. The other processes, including the mesoscale eddy-induced heat flux convergence and parameterized subgrid-scale mixing, play a secondary or negligible role. Under greenhouse warming, the COTD of mean-state SST for each EBUS is closely related to the counteraction between changes of ${Q}_{{{{{{\rm{vm}}}}}}}$ and ${Q}_{{{{{{\rm{hm}}}}}}}$ (Fig. 3e-h). The ${Q}_{{{{{{\rm{vm}}}}}}}$ change buffers the coastal warming as previous studies suggested^12–17^ except for HCS, whereas the ${Q}_{{{{{{\rm{hm}}}}}}}$ change enlarges the mean-state SST increase in the coastal region. In the Northern Hemisphere EBUSs, the enhanced cooling induced by the ${Q}_{{{{{{\rm{vm}}}}}}}$ change is dominant and responsible for the slower mean-state warming. On the contrary, the additional warming induced by the ${Q}_{{{{{{\rm{hm}}}}}}}$ change overwhelms the other processes in the Southern Hemisphere EBUSs, causing the faster mean-state warming.

**Fig. 4:**
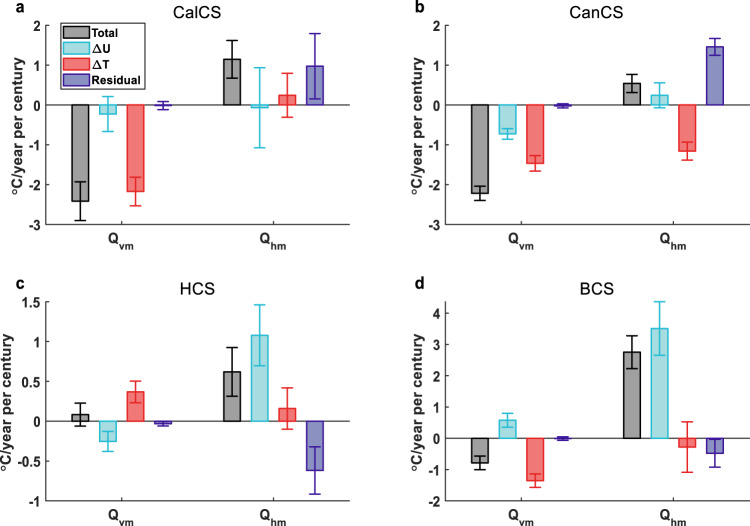
Effects of mean flow and mean-state temperature changes on mean-flow advection changes in the eastern boundary upwelling systems (EBUSs) under greenhouse warming. Decomposition of *Q*_vm_ and *Q*_hm_ changes into components associated with the mean flow changes and mean temperature gradient changes in California current system (CalCS, **a**), Canary current system (CanCS, **b**), Humboldt current system (HCS, **c**) and Benguela current system (BCS, **d**). The gray bars denote the coastal and oceanic trend difference (COTD) of mean-flow advection during 2001–2100 (the same as their counterparts in Fig. 3e–h). The blue and red bars denote the contribution by the mean flow change and mean-state temperature change, respectively; the purple bar (the residue term) denotes their interaction effects. The errorbar is the 95% confidence level. See “Heat budget analysis” in “Methods” for more computational details.

The buffering effect of upwelling on the surface EBUS warming is generally thought to be caused by the intensified upwelling in a warming climate suggested by Bakun’s hypothesis^13–16,38,39^. However, in contrast to this view, we find that the ${Q}_{{{{{{\rm{vm}}}}}}}$ change in the EBUSs is generally dominated by the change in the thermal stratification (i.e., vertical temperature gradient) rather than the upwelling strength (Fig. 4). In CalCS and BCS, the enhanced thermal stratification under greenhouse warming makes the upwelling more efficient to cool the sea surface, despite an unchanged or weakened upwelling. Both the enhanced thermal stratification and upwelling in CanCS contribute to the stronger surface cooling, but the former contribution is dominant. In HCS, the weakened thermal stratification counteracts the enhanced upwelling, so that ${Q}_{{{{{{\rm{vm}}}}}}}$ remains nearly unchanged. Therefore, the efficacy of upwelling in buffering the surface EBUS warming is largely determined by the change of thermal stratification rather than the upwelling strength in a warming climate.

The response of ${Q}_{{{{{{\rm{hm}}}}}}}$ in the Southern Hemisphere EBUSs to greenhouse warming is mainly attributed to the weakened eastern boundary currents (Fig. 4c, d; Supplementary Fig. 6a, b). Such eastern boundary current changes are largely geostrophic (Supplementary Fig. 6c, d) and robust across the high-resolution CGCM simulations in CMIP6 (Supplementary Fig. 7). The weakening of the eastern boundary current in the BCS is likely associated with a decline of the Atlantic meridional overturning circulation (AMOC)^40–42^. In addition, we find that the anthropogenic change of wind stress forcing could also contribute to the weakening of the eastern boundary currents in the Southern Hemisphere EBUSs under greenhouse warming^43^, but the underlying dynamics differ between HCS and BCS. The effect of wind stress forcing change on the weakened eastern boundary current in HCS seems largely attributed to the poleward shift of the subtropical high in the Southern Hemisphere Pacific (Supplementary Figs. 8a–d and 9)^44,45^. In contrast, the wind-induced weakening of the eastern boundary current in BCS originates in the tropical Atlantic (Supplementary Fig. 8e–h) and is likely to be related to the relaxation of trade winds associated with an Atlantic Niño-like mean-state change (Supplementary Fig. 9)^46,47^. For the Northern Hemisphere EBUSs where ${Q}_{{{{{{\rm{hm}}}}}}}$ plays a secondary role in the COTD of mean-state SST, its change is dominated by interactions between changes in the horizontal mean flows and temperature gradient, highlighting the complication in the response of coastal dynamics to greenhouse warming.

## Discussion

This study provides insight into the response of MHWs in the EBUSs to greenhouse warming and its underlying dynamics, suggesting that the Southern Hemisphere EBUSs would become local hotspots of MHWs in the future and challenging the prevailing hypothesis^12–17,21,22^ that the EBUSs serve as thermal refugia in a warming climate. So far, anthropogenic eastern boundary current changes have received less attention compared to their western boundary counterparts^48,49^. Yet our findings suggest that the weakened eastern boundary currents in the Southern Hemisphere EBUSs play a dominant role in the formation of MHW hotspots in these regions under greenhouse warming. It is thus of great importance to have an in-depth knowledge of the response of eastern boundary currents to greenhouse warming and its underlying dynamics in future studies.

**Fig. 5:**
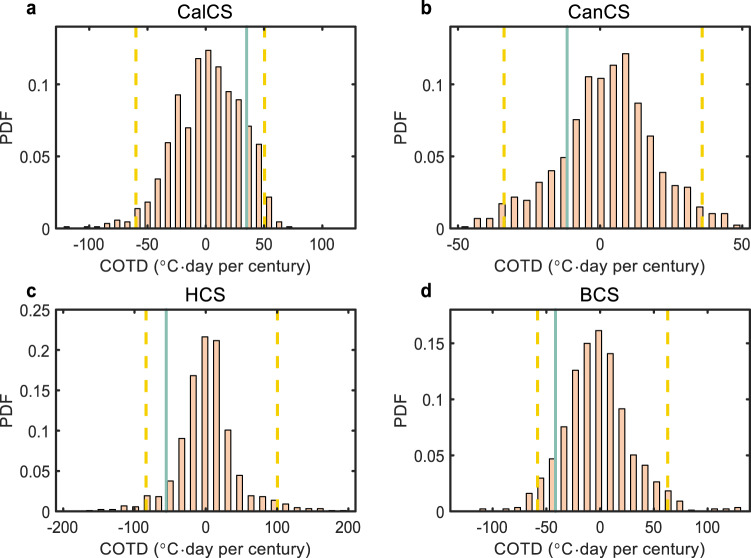
Natural multi-decadal variability of marine heatwave (MHW) stress in the eastern boundary upwelling systems (EBUSs) simulated by high-resolution coupled global climate models (CGCMs). The probability density function (PDF) of the 40-year rolling coastal and oceanic trend difference (COTD) of MHW stress averaged over California current system (CalCS, **a**), Canary current system (CanCS, **b**), Humboldt current system (HCS, **c**) and Benguela current system (BCS, **d**) in high-resolution CGCM simulations with the fixed greenhouse gas concentration. The yellow dashed lines denote the 2.5% and 97.5% percentiles. The green lines denote the COTD of MHW stress during 1982–2021 in the observation. Regions used for spatial average are delineated by the green boxes in Fig. 1a–h.

In the Southern Hemisphere EBUSs, the projected COTD of MHW stress by high-resolution CGCMs is opposite to the observed one during the past four decades (Figs. 2 and 5). This raises concerns on whether the observed multi-decadal COTD of MHW stress reflects the natural variability or whether the high-resolution CGCMs suffer from the common bias. The former seems likely as the observed multi-decadal COTD of MHW stress in all the four EBUSs lies within the range of natural variability simulated by the high-resolution CGCMs (Fig. 5). It thus implies that the forced response of MHWs in the Southern Hemisphere EBUSs to greenhouse warming has not emerged out of natural variability by now. This may be partially due to the fact that the anthropogenic decline of AMOC has not been underway^50–54^, especially for MHWs in BCS. The emergence time of anthropogenic MHW changes under different carbon emission scenarios remains unclear. Yet such knowledge would be critical for risk assessment of marine ecosystems and fisheries in the EBUSs as well as adaptation planning.

## Methods

### CESM-H

A global high-resolution climate simulation based on the Community Earth System Model (CESM-H)^35^ is used to analyze the future responses of MHWs to greenhouse warming in the four major EBUSs. The CESM-H includes an oceanic component of the Parallel Ocean Program version2 (POP2), which has a nominal horizontal resolution of 0.1° and 62 vertical levels with increasing grid space from 10 m near the surface to 250 m near 6000 m, and an atmospheric component of the Community Atmosphere Model version 5 (CAM5), which has a nominal horizontal resolution of 0.25° and 30 vertical levels with a model top at 3 hPa. Besides, it includes the Community Ice Code version 4 (CICE4) and the Community Land Model version 4 (CLM4) as its sea-ice and land components, respectively. CESM-H consists of a 500-year pre-industry control simulation (PI-CTRL) run for the 1850 condition and a 250-year historical and future transient climate run for 1850–2100 branched out from the 250th year of PI-CTRL, following the design protocol of the Coupled Model Intercomparison Project Phase 5 experiments^55^. Readers can refer to ref. 35 for a detailed description of model configurations.

### Computation of MHW stress

In this study, MHWs are identified based on the approach proposed by ref. 3, i.e., a discrete period of at least 5 consecutive days when SST is higher than a seasonally varying threshold $\theta$. The value of $\theta$ at each grid cell is computed as the 90th percentile of SST during a prescribed baseline period. The baseline period is chosen as 1950-2000 when computing the trend of MHWs during 2001–2100 under greenhouse warming but as 1982-2021 when comparing the CGCM simulated MHWs with the observations. The former period is chosen to avoid the artificial trend of MHWs due to the inhomogeneity in percentile-based indices^56^, while the latter period is chosen to be consistent with the period of observations. Once individual MHWs are identified, the MHW stress (annual cumulative intensity) is computed as the integral of MHW intensity over all the MHW days in each year^4,6,7^. To exclude the effect of long-term mean-state SST change on MHW change, we recompute MHWs by using a partial moving baseline combined with a local linear detrending following ref. 36.

### Definition of coastal and oceanic difference

The coastal and oceanic difference is defined as the value in the coastal region minus its oceanic counterpart at the same latitude. For CESM-H, the coastal region is defined as the domain within 20 model grids (~2°) from the coastline, including the coastal upwelling driven by the alongshore wind stress and a large fraction of offshore wind stress curl-driven upwelling^10,57,58^. The oceanic reference value is computed as the zonal mean value within 20-30 model grids (~2°−3°) from the coastline, where the effect of upwelling on SST becomes relatively negligible^16^. As to the CMIP6 CGCMs, their simulation data are first bilinearly interpolated onto the oceanic grids of CESM-H. The coastal and oceanic difference is then defined in the same way as in CESM-H.

### Heat budget analysis

The heat budget for the upper 50-m water column is

1 $$\frac{1}{h}\Bigg\langle {\int }_{-h}^{0}\overline{\frac{\partial T}{\partial t}}dz\Bigg\rangle=	-\frac{1}{h}\Bigg\langle {\int }_{-h}^{0}\bar{u}\frac{\partial \bar{T}}{\partial x}+\bar{v}\frac{\partial \bar{T}}{\partial y}dz\Bigg\rangle -\frac{1}{h}\Bigg\langle {\int }_{-h}^{0}\overline{w}\frac{\partial \overline{T}}{\partial z}dz\Bigg\rangle \\ 	-\frac{1}{h}\Bigg\langle {\int }_{-h}^{0}\nabla \cdot (\overline{{{{{{\bf{u}}}}}}{{{{{\boldsymbol{{{\hbox{'}}}}}}}}}T{{\hbox{'}}}})dz\Bigg\rangle+\Bigg\langle {Q}_{{{{{{\rm{shf}}}}}}}\Bigg\rangle+\Bigg\langle {Q}_{{{{{{\rm{mix}}}}}}}\Bigg\rangle$$

where $h=$ 50 m is the lower bound for the vertical integration, the angle brackets are the horizontal average over some domain (delineated by green boxes in Fig. 1a–h), T is the sea water temperature, ${{{{{\bf{u}}}}}}=(u,v,w)$ is the three-dimensional ocean velocity vector, and $\nabla=(\partial /\partial x,\partial /\partial y,\partial /\partial z)$. The overbar and prime represent the mean flows defined as the monthly mean values and mesoscale eddies defined as the perturbations from the monthly mean values, respectively.

The term on the left-hand side of Eq. (1) is the temperature tendency (denoted as TD). The first and second terms on the right-hand side are the horizontal advection (${Q}_{{{{{{\rm{hm}}}}}}}$) and vertical advection (${Q}_{{{{{{\rm{vm}}}}}}}$) by the mean flows, respectively. The third term represents the temperature flux convergence by mesoscale eddies (${Q}_{{{{{{\rm{me}}}}}}}$). The fourth term (${Q}_{{{{{{\rm{shf}}}}}}}=\frac{{\bar{F}}_{{{{{{\rm{net}}}}}}}}{{\rho }_{0}{C}_{p}h}$) measures the contribution from the sea surface heat flux with ${F}_{{{{{{\rm{net}}}}}}}$ the net sea surface heat flux defined positive into the ocean, ${\rho }_{0}$ the ocean reference density, and ${C}_{p}$ the ocean specific heat capacity. The last term (${Q}_{{{{{{\rm{mix}}}}}}}$) denotes the parameterized subgrid-scale vertical and horizontal mixing. All the terms in Eq. (1) are computed explicitly based on the CESM-H’s model output except ${Q}_{{{{{{\rm{mix}}}}}}}$ that is computed as a residue.

The effects of mean flow and mean temperature gradient changes on ${Q}_{{{{{{\rm{hm}}}}}}}$ and ${Q}_{{{{{{\rm{vm}}}}}}}$ changes under greenhouse warming can be quantified as:

2 $$\Delta {Q}_{{{{{{\rm{hm}}}}}}}=	-\frac{1}{h}\Bigg\langle {\int }_{-h}^{0}{\bar{u}}_{c}\frac{\partial \Delta \bar{T}}{\partial x}+{\bar{v}}_{c}\frac{\partial \Delta \bar{T}}{\partial y}dz\Bigg\rangle \\ 	-\frac{1}{h}\Bigg\langle {\int }_{-h}^{0}\Delta \bar{u}\frac{\partial {\bar{T}}_{c}}{\partial x}+\Delta \bar{v}\frac{\partial {\bar{T}}_{c}}{\partial y}dz\Bigg\rangle+Res.$$

3 $$\Delta {Q}_{{{{{{\rm{vm}}}}}}}=-\frac{1}{h}\Bigg\langle {\int }_{-h}^{0}{\bar{w}}_{c}\frac{\partial \Delta \bar{T}}{\partial z}dz\Bigg\rangle -\frac{1}{h}\Bigg\langle {\int }_{-h}^{0}\Delta \bar{w}\frac{\partial {\bar{T}}_{c}}{\partial z}dz\Bigg\rangle+Res.$$

where the subscript *c* represents the time mean seasonal cycle during 2001-2100 and $\Delta$ represents the difference from this time mean seasonal cycle. The first terms on the right-hand side of Eqs. (2) and (3) ($\Delta {Q}_{{{{{{\rm{hm}}}}}}}^{\Delta {T}}$ and $\Delta {Q}_{{{{{{\rm{vm}}}}}}}^{\Delta {T}}$) are caused by the change of mean temperature gradient, whereas the second terms ($\Delta {Q}_{{{{{{\rm{hm}}}}}}}^{\Delta {{{{{\bf{u}}}}}}}$ and $\Delta {Q}_{{{{{{\rm{vm}}}}}}}^{\Delta {{{{{\bf{u}}}}}}}$) are caused by the change of mean flows. The last terms, computed as a residue, are related to the cross-product of mean flow and mean temperature gradient changes.

## Supplementary information

Supplementary Information

## Data Availability

The CESM-H data used in this study are available from http://ihesp.qnlm.ac and https://ihesp.github.io/archive/products/ds_archive/Sunway_Runs.html. The CMIP6 model data used in this study can be downloaded from https://esgf-node.llnl.gov/search/cmip6/. The OISSTv2 data used in this study are provided by the NOAA (www.esrl.noaa.gov/psd/). The chlorophyll-a concentration data used in this study are obtained from https://oceandata.sci.gsfc.nasa.gov.

## Code availability

The CESM-H code is available at ZENODO via 10.5281/zenodo.3637771. The code for the computation of MHWs using a partial moving baseline combined with a local linear detrending is available at 10.5281/zenodo.7420626. The MatlabR2016b is used for plotting. Codes to reproduce the figures are available from the corresponding author upon request.
