# Supplementary material for: Southern hemisphere eastern boundary upwelling systems emerging as future marine heatwave hotspots under greenhouse warming
Source: Nat Commun. 2023 Jan 3;14:28. doi: 10.1038/s41467-022-35666-8 (PMC9810606; doi:10.1038/s41467-022-35666-8)
Supplement: Supplementary file 1 — Supplementary Information [file 41467_2022_35666_MOESM1_ESM.pdf]

Southern Hemisphere Eastern Boundary Upwelling Systems Emerging as Future Marine Heatwave Hotspots under Greenhouse Warming

Shengpeng Wang[1,2], Zhao Jing[1,2], Lixin Wu[1,2], Shantong Sun[3], Qihua Peng[4]

Hong Wang[1,2], Yu Zhang[1,2] and Jian Shi[1,5]

[1]Frontiers Science Center for Deep Ocean Multispheres and Earth System and Key Laboratory of Physical Oceanography, Ocean University of China, Qingdao, China.

[2]Laoshan Laboratory, Qingdao, China.

[3]Department of Earth, Ocean and Atmospheric Science, Florida State University, Tallahassee, Florida, USA.

[4]Scripps Institution of Oceanography, University of California San Diego, La Jolla, California， USA.

[5]College of Oceanic and Atmospheric Sciences, Ocean University of China, Qingdao, China.

List:

Supplementary Fig. 1-10

Supplementary Table 1-2

SUPPLEMENTARY INFORMATION

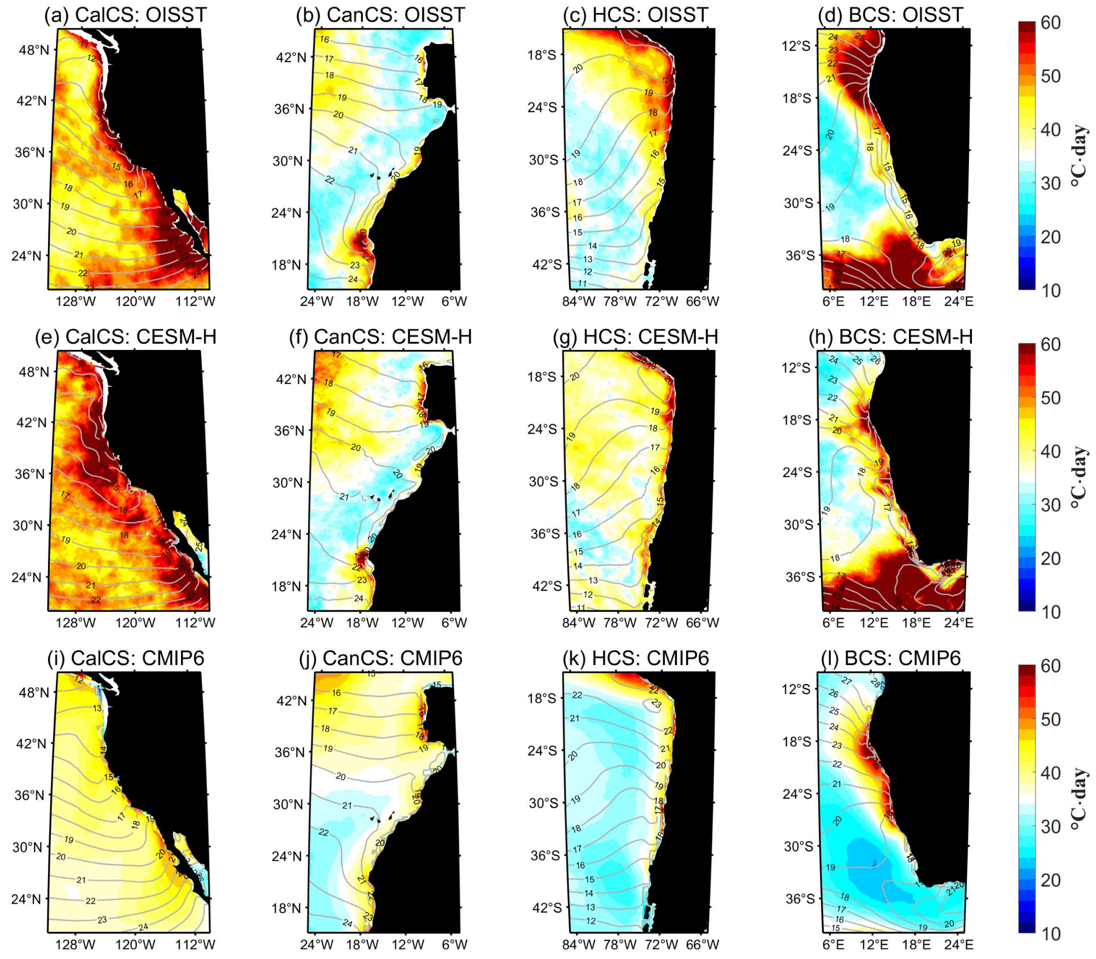

Supplementary Fig. 1 Climatological mean sea surface temperature (SST, contours) and marine heatwave (MHW) stress (shading) during 1982-2021 in the eastern boundary upwelling systems (EBUSs) obtained from the OISSTv2 product (a-d), CESM-H (e-h) and ensemble mean of low-resolution coupled global climate models in Coupled Model Intercomparison Project Phase 6 (CMIP6) (i-l).

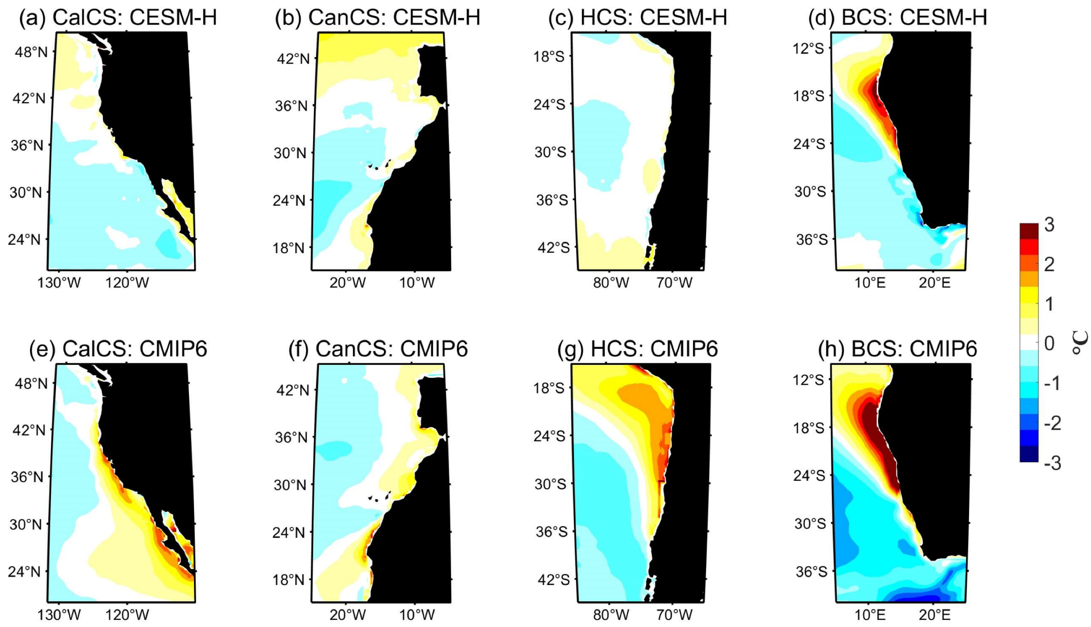

Supplementary Fig. 2 Climatological mean sea surface temperature (SST) bias during 1982-2021 in CESM-H (a-d) and ensemble mean of low-resolution coupled global climate models in Coupled Model Intercomparison Project Phase 6 (CMIP6) coupled global climate models (e-h). The bias is with respect to the observed SST (OISSTv2 product). A spatial mean SST bias over the shown regions is subtracted from each panel to highlight the SST bias related to the coastal dynamics.

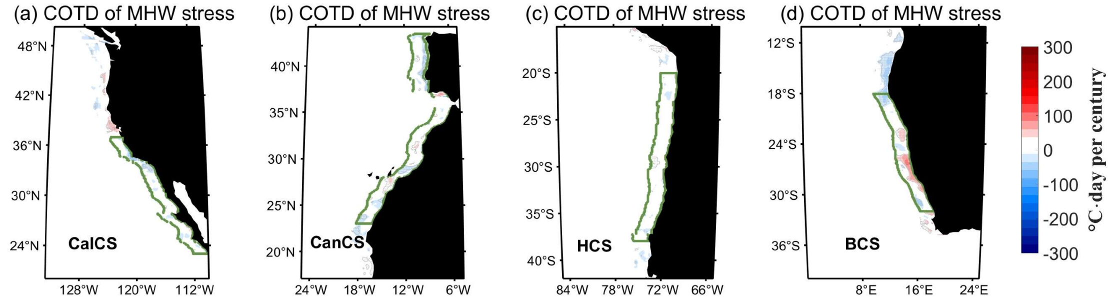

Supplementary Fig. 3 The linear trend of marine heatwave (MHW) stress during 2001-2100 in California current system (CalCS, a), Canary current system (CanCS, b), Humboldt current system (HCS, c) and Benguela current system (BCS, d) minus its counterpart in the adjacent ocean, i.e., the coastal and oceanic trend difference (COTD) of MHW stress. Here the effect of long-term mean-state sea surface temperature change on MHW change is eliminated following ref[36].

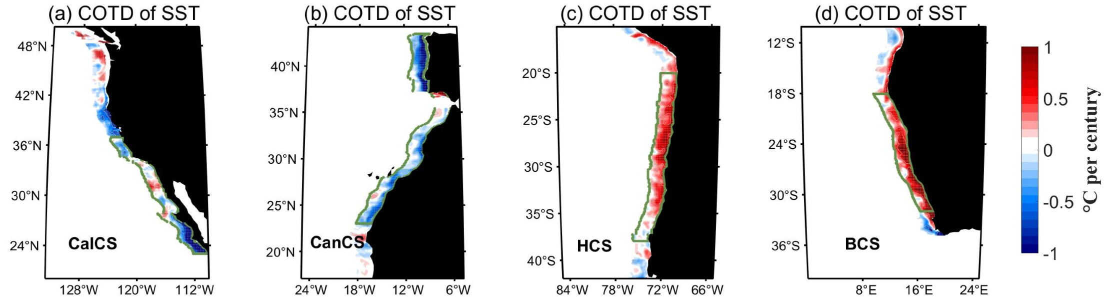

Supplementary Fig. 4 The linear trend of mean-state sea surface temperature (SST) during 2001-2050 in California current system (CalCS, a), Canary current system (CanCS, b), Humboldt current system (HCS, c) and Benguela current system (BCS, d) minus its counterpart in the adjacent ocean, i.e., the coastal and oceanic trend difference (COTD) of SST.

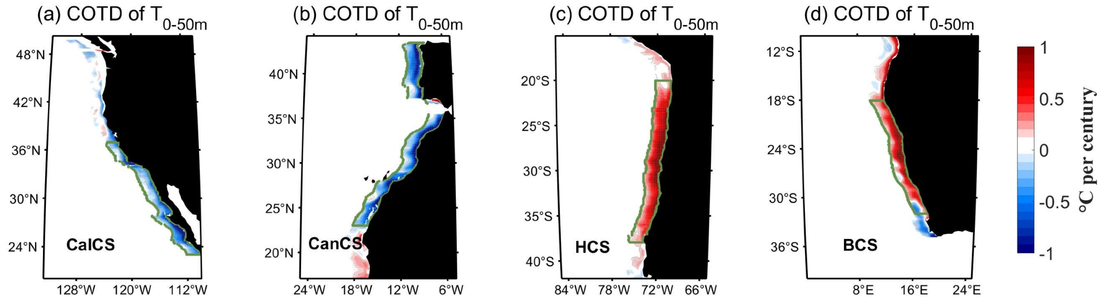

Supplementary Fig. 5 The linear trend of mean-state temperature averaged over the upper 50 m during 2001-2100 in California current system (CalCS, a), Canary current system (CanCS, b), Humboldt current system (HCS, c) and Benguela current system (BCS, d) minus its counterpart in the adjacent ocean, i.e., the coastal and oceanic trend difference (COTD) of $T_{0\text{-}50m}$.

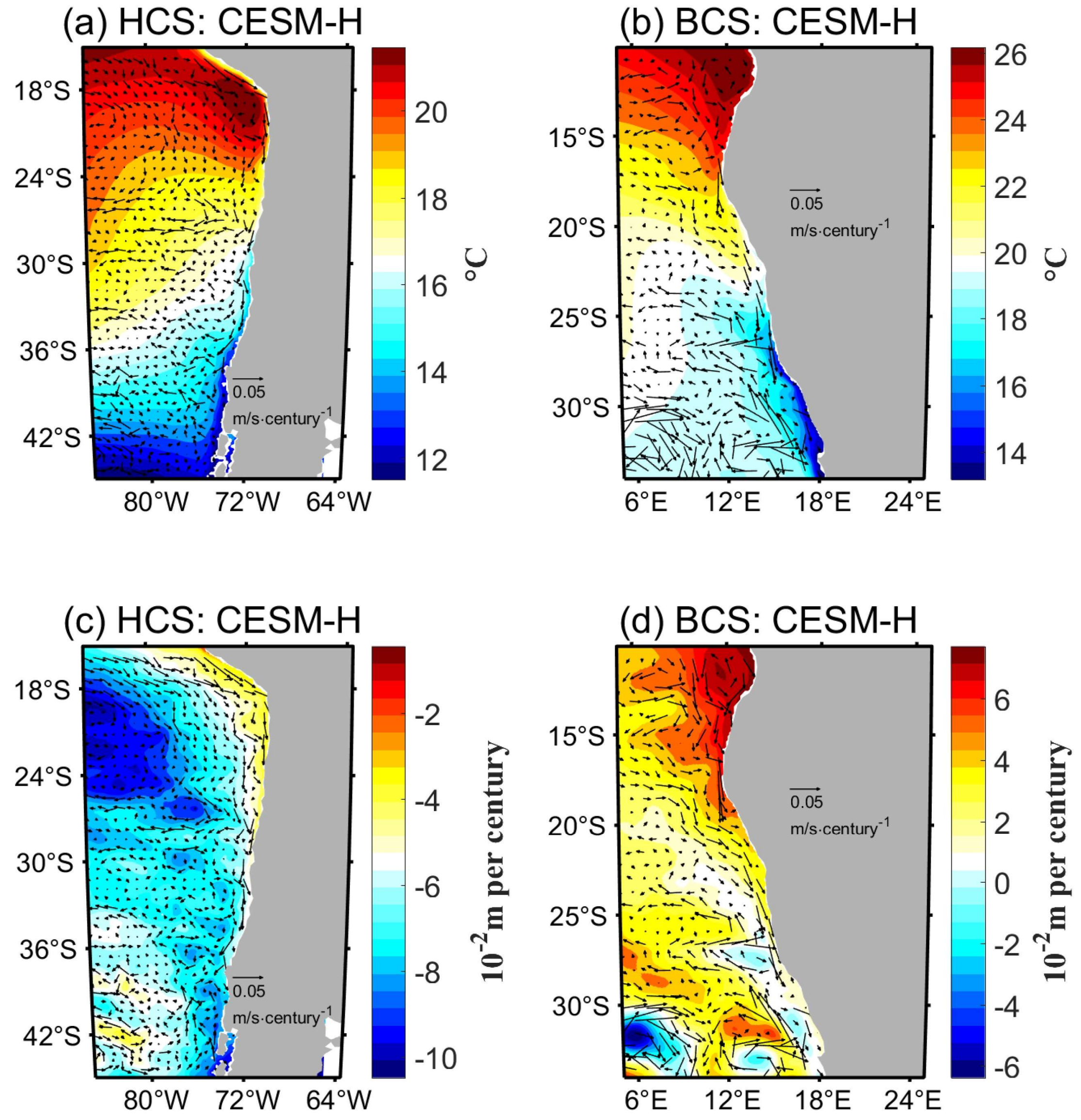

Supplementary Fig. 6 Linear trend of ocean current averaged over the upper 50 m (vector) in Humboldt current system (HCS, a) and Benguela current system (BCS, b) during 2001-2100 projected by CESM-H. Color shading is the time-mean (2001-2100) temperature averaged over the upper 50 m. Linear trends of geostrophic surface current (vector) and sea surface height (color shading) in HCS (c) and BCS (d) during 2001-2100 projected by CESM-H.

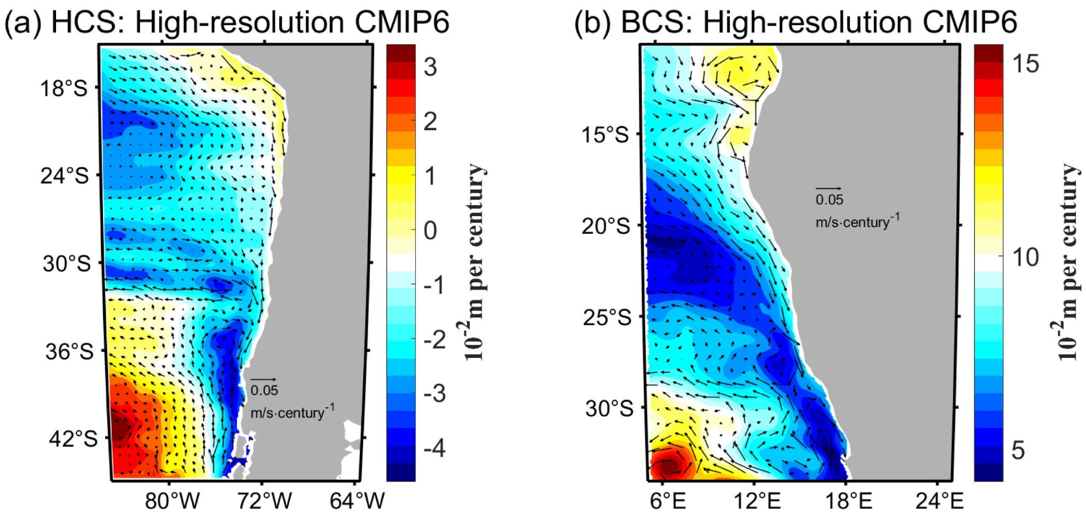

Supplementary Fig. 7 Linear trends of geostrophic surface current (vector) and sea surface height (color shading) in the Humboldt current system (HCS, a) and Benguela current system (BCS, b) projected by the ensemble mean of high-resolution coupled global climate models in Coupled Model Intercomparison Project Phase 6 (CMIP6) during 2001-2050.

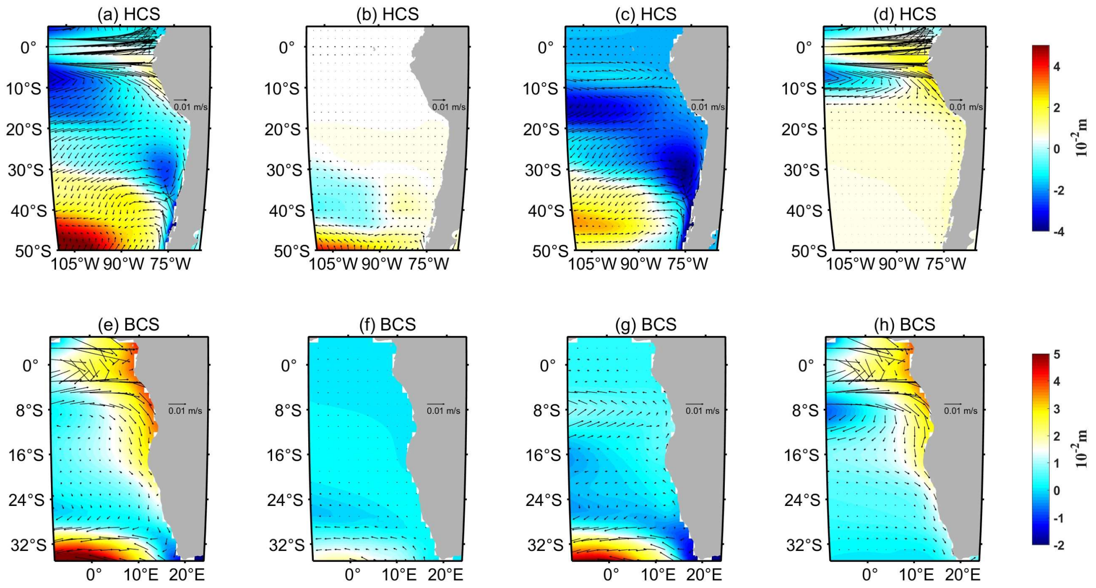

Supplementary Fig. 8 Changes of ocean current averaged over the upper 50 m (vector) and sea surface height (color shading) in the Humboldt current system (HCS, a) and Benguela current system (BCS, e) caused by the anthropogenic change of global wind stress forcing simulated by a 1° ocean general circulation model. **b,f, c,g,** and **d,h,** Same as **a,e,** but caused by the anthropogenic change of wind stress forcing in the Southern Ocean (90°S-45°S), the Southern Hemisphere subtropical gyre (45°S-8°S) and the region further northward (8°S-90°N), respectively. The model configurations are the same as those for Figure 1g of Ref[43]. The anthropogenic change of wind stress forcing is shown in Supplementary Fig. 9.

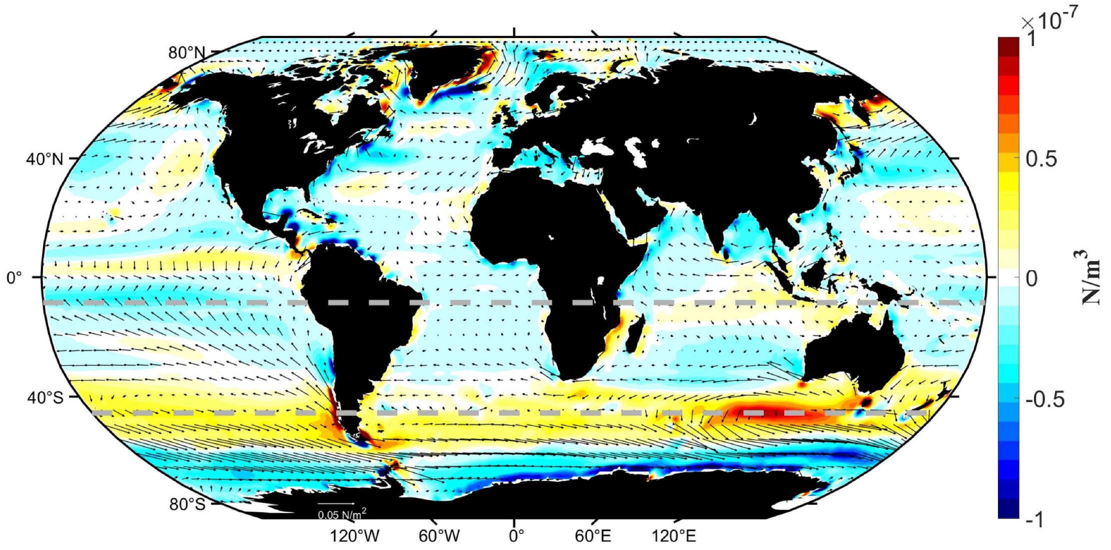

Supplementary Fig. 9 Anthropogenic changes of surface wind stress (vectors) and wind stress curl (color shading) under a high carbon emission scenario derived from the Coupled Model Intercomparison Project Phase 6 coupled global climate models (Ref[43]). The grey dashed lines divide the global ocean into the Southern Ocean (90°S-45°S), the Southern Hemisphere subtropical gyre (45°S-8°S), and the region further northward (8°S-90°N).

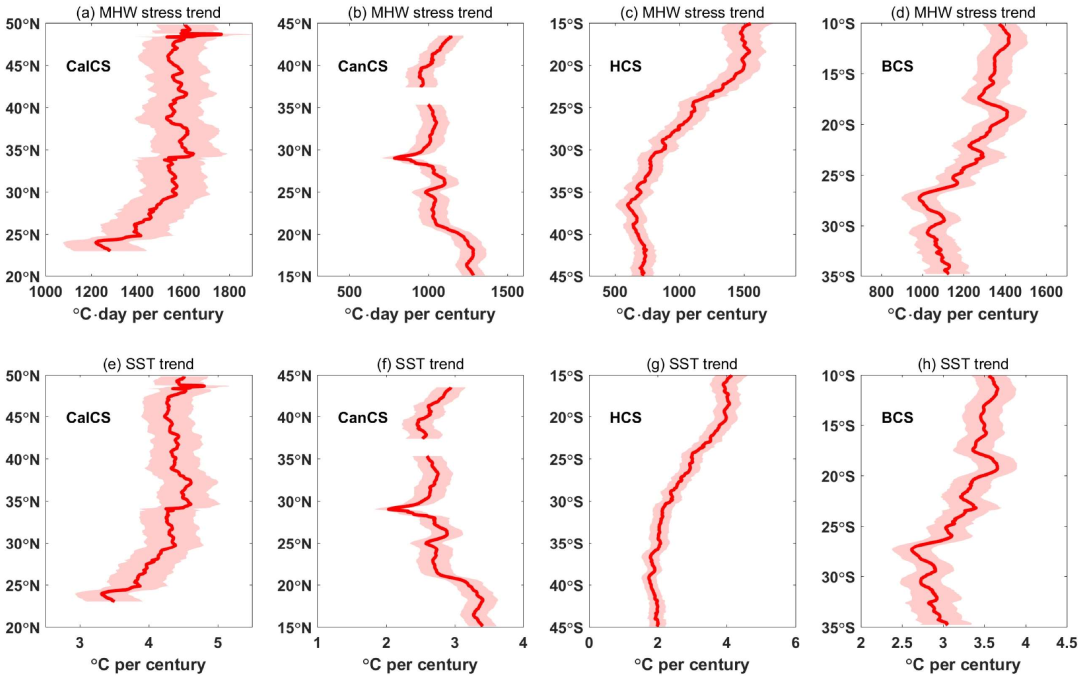

Supplementary Fig. 10 The linear trend of marine heatwave (MHW) stress during 2001-2100 in the adjacent ocean of California current system (CalCS, a), Canary current system (CanCS, b), Humboldt current system (HCS, c) and Benguela current system (BCS, d). **e-h,** Same as **a-d,** but for the annual mean sea surface temperature (SST). The shading represents the 95% confidence level.

Supplementary Table 1 A list of coupled global climate models (CGCMs) in Coupled Model Intercomparison Project Phase 6 (CMIP6). CGCMs marked in blue and red are defined as low- and high-resolution CGCMs, respectively. Each CGCM has a 251-year control simulation with the climate forcings set constantly to the 1850 condition and a 251-year historical and future transient climate simulation (1850-2100) under the high carbon emission scenario.

CGCM	Ocean Resolution	CGCM	Ocean Resolution	CGCM	Ocean Resolution
1.ACCESS-CM2	1°	10.EC-Earth3-Veg	1°	19.MPI-ESM1-2-LR	2.5°
2.ACCESS-ESM1-5	1°	11.GFDL-CM4	0.25°	20.MRI-ESM2-0	1°
3.BCC-CSM2-MR	0.5°	12.HadGEM3-GC31-LL	1°	21.NESM3	1°
4.CESM2-WACCM	1°	13.NorESM2-LM	1°	22.CMCC-CM2-SR5	1°
5.CESM2	1°	14.NorESM2-MM	1°	23.CanESM5	1°
6.CNRM-CM6-1-HR	0.25°	15. HadGEM3-GC31-MM	0.25°	24.EC-Earth3	1°
7.CNRM-CM6-1	1°	16.IPSL-CM6A-LR	1°	25.EC-Earth3-CC	1°
8.CNRM-ESM2-1	1°	17.MIROC6	1°	26.KIOST-ESM	1°
9.EC-Earth3-Veg-LR	1°	18.MPI-ESM1-2-HR	0.5°	27.UKESM1-0-LL	1°

Supplementary Table 2 A list of coupled global climate models (CGCMs) in HighresMIP of Coupled Model Intercomparison Project Phase 6 (CMIP6). Each CGCM has a 101-year control simulation with the climate forcings set constantly to the 1950 condition and a 101-year historical and future transient climate simulation (1950-2050) under the high carbon emission scenario.

CGCM	Ocean Resolution	Ensemble members	CGCM	Ocean Resolution	Ensemble members
**1.CMCC-CM2-HR2**	0.25°	**1**	**5.HadGEM3-GC31-HH**	0.1°	**1**
**2.CMCC-CM2-VHR4**	0.25°	**1**	**6.HadGEM3-GC31-HM**	0.25°	**3**
**3.CNRM-CM6-1-HR**	0.25°	**3**	**7.HadGEM3-GC31-MM**	0.25°	**3**
**4.EC-Earth3P-HR**	0.25°	**3**			
